# Pair-matched patient-reported quality of life and early oncological control following focal irreversible electroporation versus robot-assisted radical prostatectomy

**DOI:** 10.1007/s00345-018-2281-z

**Published:** 2018-03-28

**Authors:** Matthijs J. Scheltema, John I. Chang, Maret Böhm, Willemien van den Bos, Alexandar Blazevski, Ilan Gielchinsky, Anton M. F. Kalsbeek, Pim J. van Leeuwen, Tuan V. Nguyen, Theo M. de Reijke, Amila R. Siriwardana, James E. Thompson, Jean J. de la Rosette, Phillip D. Stricker

**Affiliations:** 1 Garvan Institute of Medical Research & The Kinghorn Cancer Centre, 370 Victoria Street, Sydney, NSW 2010 Australia; 2 St Vincent’s Prostate Cancer Centre, Sydney, NSW Australia; 3 Academic Medical Center, University of Amsterdam, Amsterdam, The Netherlands; 4 Erasmus Medical Center, University of Rotterdam, Rotterdam, The Netherlands; 5 UNSW, Sydney, NSW Australia; 6 School of Biomedical Engineering, University of Technology, Sydney, Australia

**Keywords:** Prostate cancer, Focal therapy, Irreversible electroporation, Radical prostatectomy, Robotic

## Abstract

**Purpose:**

The design, conduct and completion of randomized trials for curative prostate cancer (PCa) treatments are challenging. To evaluate the effect of robot-assisted radical prostatectomy (RARP) versus focal irreversible electroporation (IRE) on patient-reported quality of life (QoL) and early oncological control using propensity-scored matching.

**Methods:**

Patients with T1c–cT2b significant PCa (high-volume ISUP 1 or any 2/3) who received unifocal IRE were pair-matched to patients who received nerve-sparing RARP. Patient-reported outcomes were prospectively assessed using the Expanded Prostate Cancer Index Composite (EPIC), AUA symptom score and Short Form of Health Survey (SF-12) physical and mental components. Oncological failure was defined as biochemical recurrence (RARP) or positive follow-up biopsies (IRE). Generalized mixed-effect models were used to compare IRE and RARP.

**Results:**

50 IRE patients were matched to 50 RARP patients by propensity score. IRE was significantly superior to RARP in preserving pad-free continence (UC) and erections sufficient for intercourse (ESI). The absolute differences were 44, 21, 13, 14% for UC and 32, 46, 27, 22% for ESI at 1.5, 3, 6, and 12 months, respectively. The EPIC summary scores showed no statistically significant differences. Urinary symptoms were reduced for IRE and RARP patients at 12 months, although IRE patient initially had more complaints. IRE patients experienced more early oncological failure than RARP patients.

**Conclusions:**

These data demonstrated the superior preservation of UC and ESI with IRE compared to RARP up to 12 months after treatment. Long-term oncological data are warranted to provide ultimate proof for or against focal therapy.

**Electronic supplementary material:**

The online version of this article (10.1007/s00345-018-2281-z) contains supplementary material, which is available to authorized users.

## Introduction

Until recently no accepted curative prostate cancer (PCa) treatment had been compared in a randomized controlled trial (RCT) [1, 2]. Eight comparative RCTs were prematurely closed due to the lack of physician equipoise or patient preference [3]. The ProtecT trial was randomized between radical prostatectomy (RP), radiotherapy and active monitoring [1]. The trial recruited patients from 1999 to 2009, total estimated costs were £35 million [3]. The 10-year outcomes showed no differences in PCa-specific mortality and the patient-reported outcomes elucidated that RP had the greatest negative effect on erections sufficient for intercourse (ESI) and urinary continence (UC) [1, 4]. However, one may argue that the landscape of PCa has drastically changed since. The majority (77%) of the included patients harboured Gleason 3 + 3 = 6 on biopsy, who nowadays are most likely to have been actively monitored using improved surveillance protocols [2]. Moreover, technological advancements and experience improved the preservation of genito-urinary function following RP [5–7].

Chen et al. published an alternative method to compare prospectively acquired patient-reported quality of life (QoL) outcomes [8]. These authors compared RP, radiotherapy, brachytherapy and active surveillance using propensity score-weighted matched cohorts. Baseline characteristics were matched, whilst the cohorts had identical follow-up schemes and methods. A total of 1141 men (treated from 2011 to 2013) were enrolled in this analysis, thus providing up-to-date comparative information on QoL following different PCa treatments. This design may also be used to position new PCa treatments to the guidelines.

Focal therapy has been introduced as treatment option for patients with unifocal low-, to intermediate-risk PCa [9]. Adoption of this new treatment concept has been slow, despite increasing experience derived from phase 1–2 trials, including more than 3000 patients treated and a maximum median follow-up of 61 months [3, 9]. The lack of comparative RCTs has been advocated to be one of the main reasons. A specific PCa RCT consensus group proposed that cohort-embedded RCTs with medium-term outcomes (3–5 years) might provide proof for this concept [3].

We propose an alternative approach to compare focal therapy with current PCa treatments, by propensity score pair-matched analysis of cohorts that followed an identical follow-up scheme and evaluation. Consensus statements on focal therapy patient criteria and selection methods should be used to identify a cohort to match to an accepted PCa treatment [10, 11]. International registries may provide large cohorts to compare the long-term oncological outcomes.

In this study, we aimed to illustrate the potential of this design. Focal therapy was performed using irreversible electroporation (IRE) [12]. IRE patients, that were eligible for treatment according to the consensus guidelines [10, 11], were propensity-score matched to robot-assisted RP (RARP) patients. We evaluated the patient-reported QoL outcomes and early oncological control following focal IRE versus RARP by prospective collection of patient-reported questionnaires using identical follow-up schemes.

## Materials and methods

### Study design and patients

Patients receiving single-ablative unifocal IRE (*n* = 50) following the consensus guidelines (February 2013–July 2016) were propensity score pair-matched to patients who received nerve-sparing RARP (*n* = 325, April 2013–July 2016) [10, 11]. Eligibility criteria for both IRE and RARP patients included patients with clinical stage T1c–T2b, low-, to intermediate-risk PCa (ISUP 1–3), written informed consent for QoL evaluation, minimum of 6 months follow-up and completion of all baseline questionnaires and matching criteria (Supplementary Fig. 1). Extra eligibility criteria for RARP patients were: (1) treatment after the completion of the single-surgeons learning curve [5], (2) all procedures must be nerve-sparing. Early oncological control and prospectively collected patient-reported QoL outcomes were evaluated up to 12 months following treatment.

### Study procedures

#### Irreversible electroporation

Pre-treatment tumour localization and treatment planning were performed with transrectal or transperineal biopsies, guided by pre-biopsy mpMRI. IRE was performed by a single-surgeon (PS), including his initial learning curve and was executed following the methods as described by Ting et al. [13]. A transurethral indwelling catheter was placed to drain the bladder before treatment. The procedure was performed as a day stay procedure. The transurethral indwelling catheter was removed within 2–5 days after treatment.

#### Robot-assisted radical prostatectomy

RARP was performed by a single-surgeon (PS) after completion of his initial learning curve (> 3000 prior open RP and 1500 RARPs) [5]. The techniques described by Patel et al. [14] were followed and the Da-Vinci Xi surgical system with 6 access ports was used (Intuitive Surgical Sunnyvale^®^, CA, USA). Specifically, after the bladder neck and seminal vesicles dissection, a combined antegrade/retrograde non-thermal nerve-sparing procedure was performed. Accessory pudendal arteries were preserved. The dorsal venous plexus was dissected using electro-cautery and sutured. A suspensory suture to the symphysis pubis was placed after removal of the prostate. A two-layer Rocco stitch was used to support the posterior bladder anastomosis [15]. An anterior bladder reconstruction was performed if needed. Extended lymph node dissection was performed in cases with > 5% chance of lymph node metastasis according to the Briganti nomogram [16].

### Complication, quality of life and early oncological control evaluation

Early surgical complications were classified as specified by the Clavien–Dindo classification [17]. Patient-reported QoL outcomes were acquired at baseline, 1.5, 3, 6, and 12 months using the Expanded Prostate Cancer Index Composite (EPIC) [18], AUA symptom score [19], Short Form Health Survey (SF-12) with Physical and Mental Component Summary [20]. Clinical data managers collected the QoL questionnaires to decrease any potential impact of treating physicians. Transperineal biopsies were performed 12 months following IRE to determine early oncological control. For RARP and IRE, serial PSA testing was utilized.

### Analysis

#### Quality of life comparison

Primary analysis was performed on UC and ESI as these measures of genito-urinary function are most commonly used to report genito-urinary function outcomes following PCa treatments [6, 7, 21]. UC was defined as pad-free continence. ESI was defined as erections sufficient for intercourse with or without the use of medication. Rates of UC and ESI were compared between IRE and RARP up to 12 months, with absolute risk differences and at 12 months the number needed to treat (NNT) was calculated to prevent 1 man to develop UC or ESI. Secondary analysis included summary score differences on the questionnaires over 12 months (EPIC urinary, bowel and sexual domain, AUA symptom score, and SF-12 Physical and Mental survey). Oncological failure rates for IRE were defined by positive follow-up biopsies at 12 months with significant PCa (high-volume ISUP 1 or any 2/3). For RARP, this was defined as biochemical failure (PSA ≥ 0.2 μg/L) or the need for adjuvant radiotherapy within 12 months.

#### Matching criteria and statistical analysis

Matching was performed on age, pre-treatment PSA, ISUP score on biopsy, number of positive cores, baseline questionnaires scores and rates of pad-free UC and ESI. Propensity score matching was performed for 1:1 nearest neighbour identification. Missing data on follow-up questionnaires were not imputed and data with ≥ 1 follow-up time point were included in the comparative analysis. We employed mixed-effects models to evaluate the effect of treatment (IRE and RARP) on QoL. In this model, the outcomes (i.e., dependent variables) were EPIC urinary, sexual and bowel score, SF-12 physical and mental score, AUA score and rates of ESI and pad-free UC. Prior to the analysis, all outcome variables were re-scaled to have zero mean and unit variance (i.e., *z*-score). In this model, the standardized outcome for each individual *i* at a time point *j,* denoted by *Y*_*ij*_, is modelled as a linear function of time: *Y*_*ij*_ = π_0*j*_ + π_1*j*_ × time_*ij*_ + r_*ij*_, in which π_0*j*_ and π_1*j*_ represent the estimated baseline and rate of change for an individual, and *r*_*ij*_ is within-subject random error, which is assumed to follow a normal distribution with men 0 and variance of σ^2^. In the second level, the collection of π_0*j*_ and π_1*j*_ was then modelled as a linear function of treatment *x*_*j*_, so that the final model is: *Y*_*ij*_ = [*b*_00_ + *b*_10_ × time_*ij*_ + *b*_01_ × *X*_*j*_ + *b*_11_ × time_*ij*_ × *X*_*j*_] + [*u*_0*j *_+ *u*_1*j*_ × time_*ij*_ + *r*_*ij*_], where the terms *u*_0*j*_ and *u*_1*j*_ represent between-subject variance in the baseline estimates and between-subject variance in the rates of change, respectively. The Mann–Whitney *U* and Chi square tests were performed to assess matching characteristics. Data analysis was conducted using R including package “lme4” [22, 23].

## Results

### Patient and matching characteristics

Fifty patients treated with IRE (February 2013–July 2016) were matched to the nearest individual RARP neighbour (April 2013–July 2016) without significant matching criteria and patient characteristic differences (Table 1). Bilateral nerve-sparing RARP was performed in 94% (*n* = 47/50) of the patients. Three patients received unilateral nerve-preservation. All patients completed their baseline questionnaires. At 1.5, 3, 6, and 12 months, the response rate was 93, 97, 94 and 71% of the 100 patients, respectively. In Supplementary Fig. 1, the flowchart of the inclusion and matching is displayed.

**Table 1:** Patient and matching characteristics (median, IQR)

Variable	IRE	RARP	Match (*p* value*)*
Age (years)	67 (62–73)	67 (64–71)	Yes (*p *= 0.953)
PSA (μg/L)	5.9 (3.3–7.3)	6.3 (4.3–7.7)	Yes (*p *= 0.229)
Prostate volume (cc)	35 (30–50)	37.5 (27–50)	
Clinical stage			
T1c	37	34	
T2a	12	14	
T2b	1	2	
Biopsy (ISUP)			
1	8	9	
2	33	31	
3	9	10	
Average ISUP	2 (2–2)	2 (2–2)	Yes (*p *= 0.997)
# of cores taken	22 (10–29)	19 (14–31)	
# of positive cores	4.0 (2.8–5.3)	4.0 (3.0–7.0)	Yes (*p *= 0.189)
Baseline function			
EPIC urinary	92 (79–98)	91 (83–96)	Yes (*p *= 0.920)
EPIC sexual	65 (48–81)	69 (41–79)	Yes (*p *= 0.820)
EPIC bowel	96 (93–100)	96 (93–98)	Yes (*p *= 0.756)
SF-12 physical	56 (52–57)	55 (52–58)	Yes (*p *= 0.588)
SF-12 mental	57 (50–58)	56 (51–59)	Yes (*p *= 0.807)
AUA	6 (3–13)	7 (3–11)	Yes (*p *= 0.978)
Erections sufficient for intercourse	69% (34/49)	68% (34/50)	Yes (*p *= 0.882)
Pad-free continence rate	98% (49/50)	98% (49/50)	Yes (*p *= 1.000)

Patient characteristics are shown in median and interquartile ranges (in brackets) or in frequency. Baseline characteristics were statistically compared and a match was defined as non-statistical differences (*p* > 0.05, preferably close to *p* = 1)

### Early surgical complications

Early surgical complications for RARP included a total of nine Clavien–Dindo 1 complications and five Clavien–Dindo 2 complications (urinary retention *n* = 5, urinary tract infection *n* = 4, postoperative anemia requiring blood transfusion *n* = 1).

Eleven Clavien–Dindo 1 (mild haematuria, urgency and postoperative pain) and seven Clavien–Dindo 2 (urinary tract infection and severe postoperative pain related to the indwelling catheter) complications were reported for IRE.

### Patient-reported quality of life outcomes

**Fig. 1:**
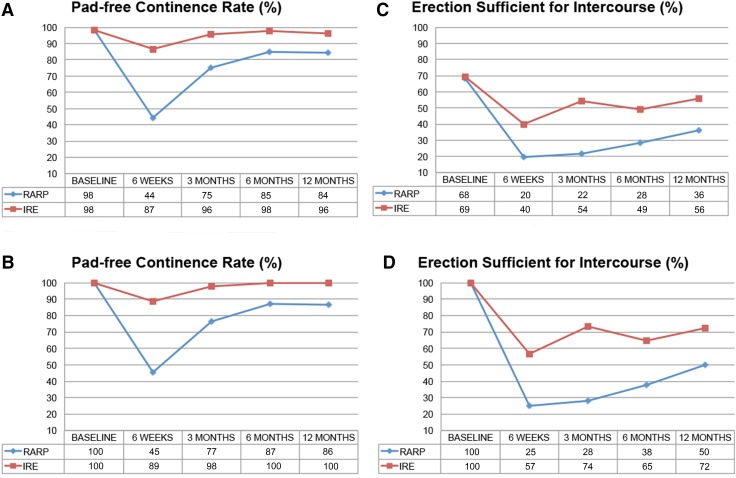
**a**, **b** Rates of pad-free urinary continence (no need for urinary pads per 24 h), for all men (**a**) and men who were continent at baseline (**b**). **c**, **d** Rates of erections sufficient for intercourse (erections firm enough to have intercourse), for all men (**c**) and men who were potent at baseline (**d**)

Baseline and absolute changes from baseline for each QoL measure, stratified by treatment group, are shown in supplementary Table 1. The results of mixed-effects model analysis are shown in Supplementary Table 2. Figure 1 illustrates the treatment outcomes on the rates of UC and ESI and Supplementary Fig. 2 the mixed-effect models on the patient-reported QoL results.

#### Urinary domain

IRE was superior to RARP in preserving pad-free UC during the first 12 months of follow-up (*p* < 0.01, Supplementary Table 2). The absolute risk reduction was 44, 21, 13 and 14% at 1.5, 3, 6, and 12 months, respectively. The number needed to treat (NNT) to preserve the UC of 1 man at 12 months was 8.3 IREs. RARP and IRE were associated with reduced urinary symptoms following treatment (AUA); however, IRE patients initially had more complaints without statistically significant difference (Supplementary Tables 1, 2). For EPIC urinary, there was a significant reduction at 6 weeks, but the scores then regressed back towards baseline levels at 3 months without significant differences between RARP and IRE or over the 12-month period (Supplementary Tables 1, 2).

#### Sexual function

IRE was superior to RARP in preserving ESI (*p* < 0.05, Supplementary Table 2) during the first 12 months of follow-up. The absolute risk reduction to develop erectile dysfunction was 32, 46, 27 and 22% at 1.5, 3, 6, and 12 months, respectively. The NNT to preserve the ESI of 1 man at 12 months was 4.5 IREs. EPIC sexual summary scores were significantly reduced for both IRE and RARP, with no differences between the two treatments (Supplementary Tables 1, 2).

#### Remaining questionnaires

No differences were found on the EPIC bowel domain and SF-12 Mental survey during the first 12 months of follow-up (Supplementary Tables 1, 2). RARP patients showed significantly more physical complaints following surgery than IRE patients on the SF-12 Physical survey at 6 weeks (Supplementary Table 1); this regressed back to baseline levels at 3 months.

#### Early oncological control

44 (88%) IRE patients underwent follow-up biopsies, five refused while one is awaiting biopsies. In total, 70.5% (31/44) men were free of significant PCa. Of those with residual significant PCa (29.5%, 13/44), five were monitored actively, three underwent salvage IRE, three salvage RARP, one salvage low-dose rate brachytherapy. One patient was diagnosed with metastatic disease directly after IRE due to persisting elevated PSA (> 10 ng/mL) that refused pre-treatment template-mapping biopsies and staging imaging. The median decline in PSA after IRE was 51% (IQR 28–85%) when the median post-IRE nadir PSA (2.8 ng/mL, IQR 0.9–4.5) was compared with the median pre-IRE PSA (5.9 ng/mL, IQR 3.3–7.3). None of the RARP patients experienced biochemical failure (PSA ≥ 0.2 ng/mL) within the first 12 months of follow-up.

## Discussion

Our results demonstrated superior rates of preserved UC and ESI with IRE compared to expert RARP in unifocal localized PCa. IRE was compared to high-standard RARP, included the first cases treated with IRE and it may be less complicated, time consuming and more economical to perform IRE. Our 12-month results are limited by large proportions of missing data, 29% versus 3–7% on other time points. Moreover, improvement of ESI and UC following RARP has been described up to 24–36 months [24]. Therefore, the 12-month follow-up of this study may underestimate the final rates of ESI and UC for both RARP and IRE patients. The outcomes of this study also showed that focal therapy is not without morbidity (e.g., 28% of potent men developed erectile dysfunction), especially in older men with poor baseline functioning. Short-term urinary complaints were more pronounced in IRE patients and were most likely due to irritation to the bladder by the extended electrical field [25]. Some of the summary scores did not reflect the different effects within their domain. For example, the EPIC urinary summary scores were not statistically different despite significant outcome differences between RARP and IRE on UC.

Patients were matched on specific parameters for QoL outcomes (e.g., baseline QoL scores), since these factors are shown to have a pronounced effect on the rates of ESI or UC following RARP [6, 7]. Interestingly, baseline genito-urinary function was not included in the randomization stratification of the ProtecT trial [4]. In the past years, the rates of UC and ESI following RARP improved remarkably compared to the RP outcomes in the ProtecT trial (12-month pad-free UC and ESI rates were 74 and 25%, respectively) [4, 6, 7, 21]. Our RARP rates of pad-free UC and ESI at 12 months (median age of 67 years) are concordant with the literature and superior to the results of our RARP learning curve analysis and the ProtecT trial [5–7].

The intent of focal therapy is to minimize collateral damage to adjacent anatomical structures by tissue-selective treatment [9]. Therefore, the genito-urinary and QoL outcomes of focal IRE must outweigh the results following RARP; as per definition, the oncological control will be inferior. In order to evaluate this equilibrium, long-term oncological data must become available. This study is limited by the absence of long-term oncological data (e.g., metastasis-free survival, overall and PCa-specific mortality). IRE patients were more likely to experience early oncological failure compared to RARP patients when using surrogates of short-term oncological control. However, it is important to highlight that this analysis included all IRE patients during the initial learning curve. Our institutional analysis showed that, when adhering to the consensus guidelines on patient selection methods, and when the IRE procedure was optimized, the oncological failure rate decreased to 13% [26, 27]. This trade-off of an increase chance for residual PCa versus improved preservation of urinary and erectile function needs to be discussed during informed consent. The potential presence of residual disease following IRE may cause psychological burden for patients. However, this seems not to apply to our cohort, as neither treatment illustrated worsening on the SF-12 mental survey. Other limitations include the retrospective analysis of prospectively acquired data, single-surgeon procedures and small cohort size.

However, the intent of this study was not to argue for a change in practice based on our short-term improved rates of UC and ESI versus the impaired surrogates of oncological control following IRE. We aimed to address the need for a new trial design to compare curative PCa treatments, show the feasibility of this propensity-score matching design and validate the rationale of focal therapy. The highest level of unbiased data is attained by the use of head-to-head multi-centre randomized trials, including large cohorts and long-term follow-up. In comparison, propensity score pair-matched cohorts are inherently more prone to be biased. Nevertheless, one may argue that the ever-evolving PCa therapy landscape, biological nature of the disease and strong patient preference hinder the ability to draw firm conclusions from long-term randomized trials. Carefully matched cohorts with long-term QoL and oncological outcome data derived from international registries have the potential to approximate the strength of randomized trials. To reach this goal, it will be crucial to establish the use of identical follow-up methods, time points and questionnaires throughout different treatments. This may create an open platform that may compare new PCa treatments affordably and efficiently to the current accepted segments.

## Electronic supplementary material

Below is the link to the electronic supplementary material.

Supplementary figure 1 The flowchart of the patient inclusion and matching. Supplementary material 1 (TIFF 6248 kb)

Supplementary figure 2 Legend: The outcomes of the mixed-effects models per questionnaire. By use of mixed-effects models the standardized outcome for each individual was modelled (individual lines), followed by the collective outcomes per treatment (marked line). The 95% confidence intervals are illustrated in grey. Supplementary material 2 (TIFF 51389 kb)

Supplementary material 3 (DOCX 16 kb)
