# Supplementary figures and images for: Pair-matched patient-reported quality of life and early oncological control following focal irreversible electroporation versus robot-assisted radical prostatectomy
Source: World J Urol. 2018 Mar 28;36(9):1383–9. doi: 10.1007/s00345-018-2281-z (PMC6105143; doi:10.1007/s00345-018-2281-z)

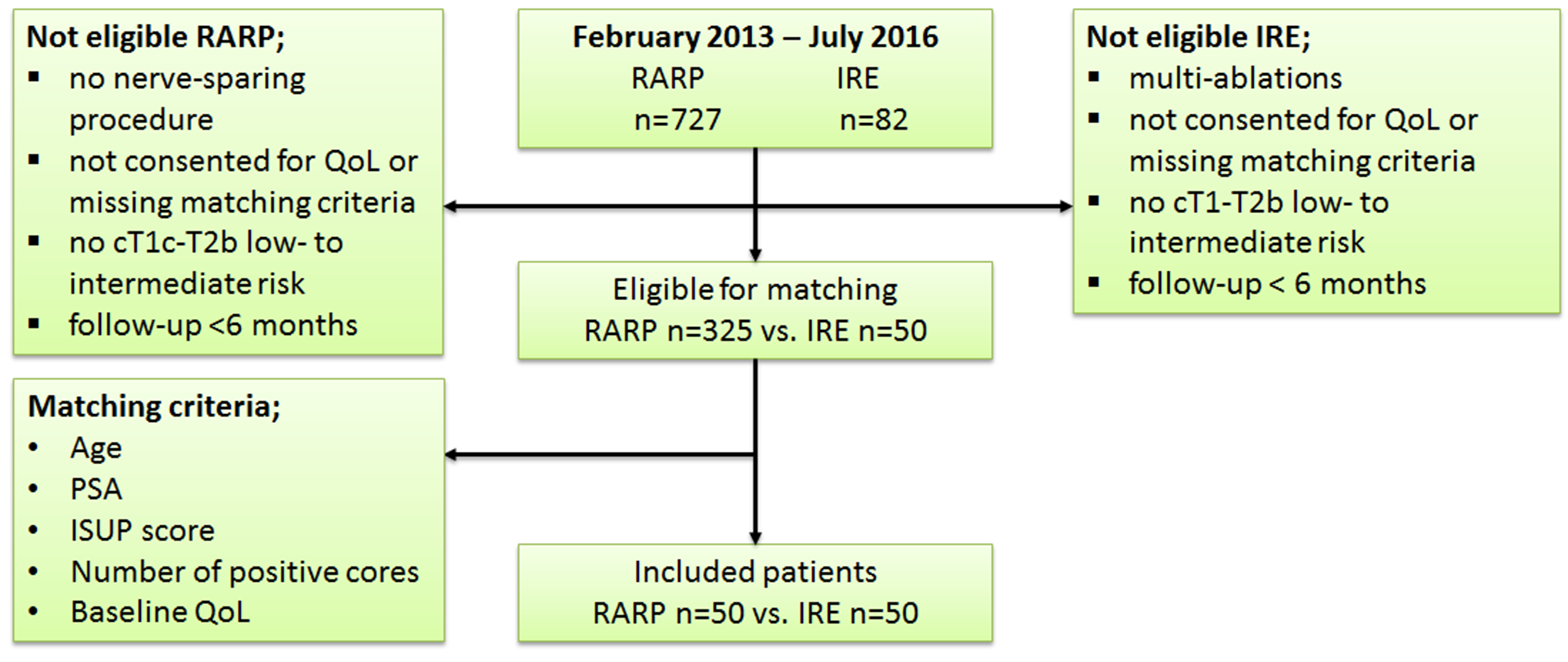

Supplement: Supplementary file 1 — Supplementary figure 1 The flowchart of the patient inclusion and matching. Supplementary material 1 (TIFF 6248 kb) [file 345_2018_2281_MOESM1_ESM.tif]
