# Supplementary material for: Pair-matched patient-reported quality of life and early oncological control following focal irreversible electroporation versus robot-assisted radical prostatectomy
Source: World J Urol. 2018 Mar 28;36(9):1383–9. doi: 10.1007/s00345-018-2281-z (PMC6105143; doi:10.1007/s00345-018-2281-z)
Supplement: Supplementary file 3 — Supplementary material 3 (DOCX 16 kb) [file 345_2018_2281_MOESM3_ESM.docx]

**Supplementary table 1: Summary of quality of life parameters at baseline and follow-up periods grouped by treatment**

| **Variable** | **Treatment** | **Baseline** | **6 weeks** | **3 months** | **6 months** | **12 months** |
| --- | --- | --- | --- | --- | --- | --- |
| Urinary | IRE | 87.6 (11.2) | -14.5 (16.9) | -0.4 (11.2) | 1.4 (11.7) | 1.1 (9.5) |
|  | RARP | 87.6 (11.4) | -15.0 (14.6) | -2.2 (11.1) | 0.7 (10.5) | 1.3 (10.0) |
| Bowel |  |  |  |  |  |  |
|  | IRE | 93.6 (8.9) | -1.2 (9.2) | -0.5 (13.1) | 0.3 (7.8) | 0.2 (12.1) |
|  | RARP | 94.5 (5.6) | -2.6 (9.8) | -0.6 (7.3) | 0.1 (7.7) | -0.1 (6.9) |
| Sexual |  |  |  |  |  |  |
|  | IRE | 62.8 (23.4) | -18.1 (19.1) | -12.2 (18.5) | -11.1 (14.9) | -15.4 (14.3) |
|  | RARP | 61.9 (23.9) | -25.7 (27.6) | -23.6 (23.8) | -21.1 (25.5) | -17.0 (22.0) |
| SF12 PCS |  |  |  |  |  |  |
|  | IRE | 53.4 (5.8) | -1.3 (3.8) | -0.1 (4.1) | -0.5 (6.2) | -1.5 (6.5) |
|  | RARP | 54.1 (5.4) | -9.7 (7.9) | -1.8 (7.4) | -1.4 (8.5) | -0.5 (7.3) |
| SF12 MCS |  |  |  |  |  |  |
|  | IRE | 52.4 (10.4) | 1.6 (9.1) | 0.1 (6.7) | -2.1 (7.2) | -0.0 (10.9) |
|  | RARP | 53.4 (8.2) | 1.4 (8.6) | 0.6 (7.2) | -1.0 (9.4) | 0.2 (9.0) |
| AUA |  |  |  |  |  |  |
|  | IRE | 7.9 (6.6) | 1.5 (6.1) | -0.7 (5.9) | -0.9 (4.3) | -0.5 (5.2) |
|  | RARP | 7.8 (6.4) | -0.9 (4.9) | -3.5 (5.7) | -3.7 (6.1) | -3.4 (6.0) |

**Legend**: Baseline data are shown in mean and standard deviation (in brackets). For 6-week, 3-month, 6-month and 12-month data are shown in absolute change from baseline and standard deviation.

**Supplementary table 2. Effects of treatment on measures of quality of life: results of mixed-effects model analysis**

| **Parameter** | **Urinary** | **Bowel** | **Sexual** | **SF12-PCS** | **SF12-MCS** |
| --- | --- | --- | --- | --- | --- |
| **Fixed effects** | | | | | |
| Time | 0.118 (0.038) | -0.008 (0.041) | **-0.087 (0.030)** | -0.028 (0.042) | -0.032 (0.033) |
| Treatment (RARP vs IRE) | -0.032 (0.208) | -0.018 (0.189) | -0.157 (0.181) | **-0.467 (0.189)** | 0.048 (0.195) |
| Time x Treatment | -0.003 (0.051) | 0.039 (0.054) | -0.043 (0.040) | 0.096 (0.056) | 0.025 (0.045) |
| **Variance components** | | | | | |
| Between subjects | 0.448 (0.669) | 0.195 (0.441) | 0.441 (0.664) | 0.162 (0.402) | 0.539 (0.734) |
| Rates of change | 0.000 (0.000) | 0.004 (0.067) | 0.002 (0.047) | 0.007 (0.085) | 0.008 (0.091) |
| Within subjects | 0.525 (0.724) | 0.579 (0.761) | 0.302 (0.549) | 0.568 (0.753) | 0.332 (0.576) |

| **Parameter** | **AUA** | **Continence (all)** | **Continence (continent)** | **Potency (all)** | **Potency (potent)** |
| --- | --- | --- | --- | --- | --- |
| **Fixed effects** |  |  |  |  |  |
| Time | **-0.092 (0.038)** | **-0.982 (0.424)** | **-0.872 (0.423)** | 0.028 (0.375) | -0.512 (0.460) |
| Treatment (RARP vs IRE) | -0.152 (0.246) | **1.698 (0.636)** | **1.805 (0.661)** | 0.308 (0.448) | 0.513 (0.534) |
| Time x Treatment | -0.085 (0.052) | 0.533 (0.402) | 0.481 (0.414) | **1.437 (0.627)** | **1.664 (0.695)** |
| **Variance components** |  |  |  |  |  |
| Between subjects | 1.126 (1.061) |  |  |  |  |
| Rates of change | 0.028 (0.168) |  |  |  |  |
| Within subjects | 0.322 (0.567) |  |  |  |  |

**Legend**: All numbers represent regression coefficients and standard error (in brackets) for an effect over time, between treatments and an overall effect over time between treatments, including the variance components. The effects with statistical significance (p<0.01) are shown in bold-faced numbers.
